# Factors Contributing to Successful Information System Implementation and Employee Well-Being in Health Care and Social Welfare Professionals: Comparative Cross-Sectional Study

**DOI:** 10.2196/52817

**Published:** 2024-11-21

**Authors:** Janna Nadav, Anu-Marja Kaihlanen, Sari Kujala, Ilmo Keskimäki, Johanna Viitanen, Samuel Salovaara, Petra Saukkonen, Jukka Vänskä, Tuulikki Vehko, Tarja Heponiemi

**Affiliations:** 1 Department of Healthcare and Social Welfare, Finnish Institute for Health and Welfare, Mannerheimintie 166, Helsinki, 00271, Finland, 358 29 524 6000; 2 Faculty of Social Sciences, Tampere University, Helsinki, Finland; 3 Department of Computer Science, Aalto University, Espoo, Finland; 4 Faculty of Social Sciences, University of Lapland, Rovaniemi, Finland; 5 The Finnish Medical Association, Helsinki, Finland

**Keywords:** implementation, health information systems, client information systems, stress, healthcare professionals, social welfare professionals, clinician well-being, workplace stress

## Abstract

**Background:**

The integration of information systems in health care and social welfare organizations has brought significant changes in patient and client care. This integration is expected to offer numerous benefits, but simultaneously the implementation of health information systems and client information systems can also introduce added stress due to the increased time and effort required by professionals.

**Objective:**

This study aimed to examine whether professional groups and the factors that contribute to successful implementation (participation in information systems development and satisfaction with software providers’ development work) are associated with the well-being of health care and social welfare professionals.

**Methods:**

Data were obtained from 3 national cross-sectional surveys (n=9240), which were carried out among Finnish health care and social welfare professionals (registered nurses, physicians, and social welfare professionals) in 2020‐2021. Self-rated stress and stress related to information systems were used as indicators of well-being. Analyses were conducted using linear and logistic regression analysis.

**Results:**

Registered nurses were more likely to experience self-rated stress than physicians (odds ratio [OR] –0.47*; P*>.001) and social welfare professionals (OR –0.68; *P*<.001). They also had a higher likelihood of stress related to information systems than physicians (b=–.11; *P*<.001)*.* Stress related to information systems was less prevalent among professionals who did not participate in information systems development work (b=–.14; *P*<.001)*.* Higher satisfaction with software providers’ development work was associated with a lower likelihood of self-rated stress (OR –0.23*; P*<.001) and stress related to information systems (b=–.36 *P*<.001)*.* When comparing the professional groups, we found that physicians who were satisfied with software providers’ development work had a significantly lower likelihood of stress related to information systems (b=–.12; *P*<.001) compared with registered nurses and social welfare professionals.

**Conclusions:**

Organizations can enhance the well-being of professionals and improve the successful implementation of information systems by actively soliciting and incorporating professional feedback, dedicating time for information systems development, fostering collaboration with software providers, and addressing the unique needs of different professional groups.

## Introduction

The integration of information systems (ISs) into health care and social welfare organizations has prompted significant changes in patient and client work [1]. The integration and implementation of these systems are expected to provide many benefits for professionals, such as better documentation, decision-making, and information management [2-4]. However, it is noteworthy that while health information systems (HISs) and client information systems (CISs) offer many advantages to health care and social welfare professionals (SWPs), their implementation can also introduce additional stress due to the increased time and effort required from the professionals, which reduces the time available for direct patient or client care [1,5-8]. Furthermore, there are instances where professionals struggle to use the established systems [9-11], receive insufficient training or support from the organizations [5,6,9,12-14], or must constantly adjust to new working methods because of new systems. [15,16].

Health care and SWPs work is often burdensome, and the COVID-19 pandemic placed an additional strain on them and challenged their ability to cope with everyday work [17]. Stress among these professionals has been growing in recent years for reasons such as increasing workload [18,19], staff shortage [20-22], shift work and irregular hours [23,24], and emotional and psychological challenges in patient or client work [25-27]. Consequently, poorly functioning and constantly changing ISs may increase professionals’ stress even more [11,28,29].

Stress related to information systems (SRIS) among health care professionals has been a subject of interest in recent years [5,7,28,30-34]. A longitudinal study by Heponiemi et al [28] found that SRIS had increased progressively among Finnish physicians. Poor usability of HISs has been found to be associated with physicians’ stress [11]. Also, registered nurses (RNs) experience higher levels of SRIS when using HIS with poor usability [7] or if they have experienced an HIS implementation recently [5]. Furthermore, SRIS has previously been found to be associated with time pressure, cognitive workload, and high levels of technical problems related to the HIS among physicians and RNs [5,9,28,29]. Among SWPs, this kind of research has been conducted relatively rarely.

It has been suggested that end user participation in IS development is central [35], as it may improve the success of the implementation and even have a positive association with professionals’ well-being [36,37]. Based on Finnish national surveys, RNs, physicians, and SWPs are willing to participate in IS development [37-39]. However, there seems to be a lack of appropriate methods to effectively involve them and incorporate their feedback into the development work [37,38]. According to Martikainen et al [38], one of the reasons for RNs’, physicians’, and SWPs’ reluctance to participate in the development is that they do not have working time allocated for it. In addition, earlier negative experiences, for example, not being able to influence the development in their preferred ways, may reduce professionals’ willingness to prioritize their time for IS development work [38]. In addition, because CISs in social welfare have often been developed from administrative perspectives [40-44], professionals in the field may feel that their opportunities to influence are weak. This may lead to a situation where professionals are cautious about the following implementations and reluctant to participate in the development work [40,41].

Being able to give feedback about the ISs is important for professionals, as it gives them the feeling that they can have an influence [6]. Furthermore, it may also give the software providers an opportunity to further develop the systems based on the professionals’ needs. Studies conducted in Finland have identified a significant gap between the perceptions of software developers and the experiences of health care professionals and SWPs concerning user feedback and its integration into software development processes [35,37,38,45]. While a significant majority of developers (90%) expressed interest in user feedback, with 81% claiming to consider end users’ opinions and experiences during software development, professionals, including nurses, physicians, and SWPs, reported a different reality [37,38]. Only half of the professionals knew who to contact and how to provide feedback, and a mere few believed that vendors were genuinely interested in end users’ viewpoints or that corrections and change requests were implemented according to their suggestions or within a reasonable time frame [35,37,38,45]. Furthermore, an interview study among health care and SWPs found that it is central to the professionals that everyone knows where to give feedback and that it is acted upon [6]. If the feedback process regarding IS development does not work smoothly, it has been shown to affect physicians’ well-being by increasing their stress [9].

Although there are some studies that have examined the well-being of health care professionals in relation to HISs, they have mainly focused on one professional group at a time and more on the IS usability perspective [7,9,30]. However, little research addresses health care professionals’ and SWPs’ well-being in relation to the factors that can contribute to the success of implementations. Moreover, few studies, if any, have compared these professional groups in this setting. Therefore, we aimed to examine whether the factors that contribute to the successful implementation of information systems (participation in HIS or CIS development and satisfaction with software providers’ development work) are associated with well-being among RNs, physicians, and SWPs. More specifically, we aimed to examine whether there are differences in potential associations between these professional groups. It is important to study these professional groups, because they are key users of ISs in the Finnish health care and social welfare field, and their daily workflow is directly impacted by the efficiency and usability of these systems [46]. By examining and comparing the well-being of these professional groups, we may get valuable insights into the unique challenges and stressors faced by each group and identify potential areas for improvement in IS development work.

The specific research questions were as follows: (1) Are professional groups, participation in HIS or CIS development, and satisfaction with software providers’ development work associated with self-rated stress and SRIS? (2) Are there differences between professional groups in the associations of (a) participation in HIS or CIS development, and (b) satisfaction with software providers’ development work with self-rated stress and SRIS?

## Methods

### Sample

The data were collected through online surveys [47] among RNs, public health nurses, and midwives (with at least bachelor-level education and younger than the age of 65 years) during March and April 2020, among SWPs (with at least bachelor-level education; eg, social workers and social counselors and younger than the age of 65 years) during September and October 2020, and among physicians (licensed physicians younger than 65 years) during January and March 2021.

The RNs’ contact information were collected by the Finnish Nurses Association, Tehy (the union of health and social care professionals in Finland), and the National Professional Association for the interests of experts and managers in health care (TAJA; n=29,283). The SWPs’ contact information were collected by the Union of Professional Social Workers (Talentia), the Trade Union for the Public and Welfare Sectors (JHL), and Social Science Professionals (YKA; n=12,471). The contact information of physicians were collected by the Finnish Medical Association (n=19,142). Invitations to participate in the questionnaire were sent via e-mail to professionals who were registered members of the abovementioned trade unions and professional associations. For RNs, 1 reminder was sent, while physicians received 3 reminders. Notably, for SWPs, the survey link was also disseminated through social media when the response rate was at a risk to remain low [37].

The collected contact information from the abovementioned trade unions and professional associations represents a substantial portion of the respective professional populations in Finland, comprising 47.23% (29,283/62000) of all working-age RNs, 40.23% (12,471/31,000) of all working-age SWPs, and 90.51% (19,142/21,148) of all working-age physicians [39,48]. Detailed description of the data collection from RNs [5,39,49], physicians [39,50], and SWPs [39,51] can be found elsewhere. The final sample for this study included 9240 respondents: 3610 RNs (with a 12% response rate), 4640 physicians (with a 24% response rate), and 990 SWPs (with 8% response rate). The response rate for SWPs could not be precisely calculated because the survey link was also distributed through social media.

### Ethical Considerations

Participation in the surveys was voluntary, and all participants provided informed consent by actively engaging in the study through questionnaire responses. Ethical approval for the questionnaires used with nurses and SWPs was applied from the Finnish Institute for Health and Welfare (THL/482/6.02.01/2020). For the physician’s questionnaire, an ethics committee statement was not applied. This decision aligns with the guidelines outlined by the Finnish National Board on Research Integrity [52], as surveying respondents for their opinions without anticipated harm does not require an ethics committee statement.

### Context

In Finland, during the data collection, the municipalities were responsible for organizing health and social services [53]. However, a substantial structural change took place in January 2023 with the creation of 22 well-being service counties (WBSCs). This change reflects a decisive move toward integrating health and social services, emphasizing a commitment to offering more coordinated, person-centered, and sustainable services. The primary goal of this integration is to eliminate historical divisions between health and social services, promoting a seamless and comprehensive approach to well-being for all residents [54]. The WBSCs are responsible for organizing primary care, secondary health care, and social and rescue services for their residents [55]. These counties are governed by elected councils and are funded through the state budget. Professionals working in the public sector typically receive fixed budget-based salaries in these counties. The WBSCs have the flexibility to provide services independently or in collaboration with other WBSCs [55,56].

Finland is one of the leading countries in digitalization [57], and along with the other Nordic countries has made considerable progress in the development and implementation of HISs and CISs in the health care and social welfare field [39]. For example, health care professionals’ access to HISs is 100% in both public and private sectors, and access to CISs in social welfare is nearly 100% in the public sector and 75% in the private sector [58,59]. There are more than 20 HIS and CIS brands used in the health care and social welfare field in Finland [60]. The Finnish Ministry of Social Affairs and Health consistently initiated national surveys focused on monitoring the status and emerging trends in the realm of electronic health and electronic welfare services in Finland. The primary goal was to gather empirical data that inform the ongoing development of these sectors [61].

### Measurements

The well-being of professionals was measured using 2 stress-related indicators: self-rated stress and SRIS.

Self-rated stress was measured with a validated single-item measure stating that “Stress refers to a situation in which a person feels tense, restless, nervous, or anxious, or finds it hard to sleep because of constantly worrying about things,” and asking whether the respondent was feeling this kind of stress nowadays [62]. This variable was rated on a 5-point scale ranging from 1 (not at all) to 5 (very much). For the analyses, responses were coded into 2 categories (0=no stress and 1=stressed), so that the original options 1‐2 were “no stress” and options 3‐5 were “stressed.”

SRIS was measured with two items asking how often (1) changing information systems or (2) awkward or poorly functioning systems have disturbed, worried, or burdened professionals’ work during the past 6 months. In the SWPs’ questionnaires, the items were rated on a 5-point Likert scale and the options ranged from 1 (very rarely or never) to 5 (very often or constantly). However, in the physicians’ questionnaire, the options were in the opposite order and were therefore reverse-coded. In the RNs’ questionnaire, the items were rated on a 6-point Likert scale, and the options ranged from 1 (never) to 6 (constantly). The items were combined into a mean sum variable (Cronbach α=0.74). The mean scores were standardized with *z* scores to enable comparability (explained in more detail in the Statistical Analyses section). This measure has been developed in Finland and it has been used among physicians and RNs and associated, for example, with time pressure, cognitive workload, and high levels of technical problems of the HIS [eg, 5,9,14,28,29].

The factors contributing to the success of the implementation were measured using 2 measures, which were participation in HIS or CIS development and satisfaction with software providers’ development work.

Participation in HIS or CIS development was assessed by asking whether the respondent participated in HIS or CIS development work. There were three response options: (1) “yes, some of my working time has been allocated for such development work,” (2) ”yes, in addition to my work,” and (3) “no.”

Satisfaction with software providers’ development work was assessed using 3 statements from the validated National Usability-Focused HIS Scale [3]. Respondents were asked to assess three statements based on their experience: (1) the software provider is interested in end users’ feedback about the system, (2) the software provider implements corrections and change requests according to the suggestions of the end users, and (3) corrections and change requests are implemented within a reasonable time frame. The answer options in the physicians’ and SWPs’ questionnaires ranged from 1 (fully agree) to 5 (fully disagree). However, in the nurses’ questionnaire, the options ranged from 1 (fully agree) to 5 (disagree) and additionally an option 6 (I cannot say), which was coded as a missing value. These items were reverse coded to ensure more logical data analysis and then combined into a mean sum variable (Cronbach α=0.89).

Respondents’ gender (1=women, 2=men, 3=other or do not want to say), age (1=<35, 2=35‐44, 3=45‐54, and 4=>54 years), and working sector (1=public, 2=private/other) were also requested in the survey and coded similarly. These adjustment variables were used because they have played an important role in professionals’ well-being in previous studies [eg, 1,11,30]. Measures used in this study can be found in the Multimedia Appendix 1.

### Statistical Analyses

Because the response scales in the RNs’ questionnaire differed from the physicians’ and SWPs’ questionnaires, the means of SRIS were standardized with *z* scores. The *z* score describes how many SDs you are away from the mean (mean always=0 and SD always=1). The *z* score is positive if the value is higher than the mean, and negative if lower than the mean [63]. This allowed us to make comparisons when we merged the data [64]. Before the analysis, we used the multiple imputation method in R statistical software to impute the missing data [65]. All the study variables were included in the imputation models and the datasets were imputed 5 times [66,67].

Logistic regression analyses were conducted with professional groups, participation in HIS or CIS development, and satisfaction with software providers’ development work as independent variables and self-rated stress as the dependent variable (binarily coded). Linear regression analyses were used to examine the associations of the same abovementioned independent variables with SRIS (continuous dependent variable). In addition, we tested that the data met the assumptions of linearity, homoscedasticity, multicollinearity, and that the residuals were approximately normally distributed. Analyses of the main effects were conducted in 2 steps. First, the univariable effects were examined. Second, the fully adjusted model included the professional group, participation in HIS or CIS development, satisfaction with software providers’ development work, and the adjustment variables age, gender, and working sector.

In addition, we examined the potential interactions of the professional group with (1) participation in HIS or CIS development and (2) satisfaction with software providers’ development work (in separate analyses) for stress and SRIS. These analyses were also adjusted for age, gender, and working sector. All the analyses were conducted using RStudio (version 4.1.1; R Core Team 2020).

## Results

### Characteristics of the Professional Groups

The characteristics of the respondents are presented in Table 1. Most of the respondents were women (RNs: 93% [3340/3610]; physicians: 64% [2978/4640]; and SWPs: 92% [913/990]) and worked in the public sector (RNs: 86% [3115/3610]; physicians: 80% [3698/4640]; and SWPs: 86% [846/990]), which corresponds to the general gender and employment structure of these professionals in Finland [68]. Almost 25% of the RNs (796/3610) and physicians (1116/4640) had participated in HIS development, whereas 33% (326/990) of SWPs had participated in CIS development.

**Table 1.** Characteristics of the professional groups.

Characteristics		All (N=9240)	RNs^a^ (n=3610)	Physicians (n=4640)	SWPs^b^ (n=990)
**Gender, n (%)**					
	Women	7231 (78.3)	3340 (92.5)	2978 (64.2)	913 (92.2)
	Men	1934 (20.9)	249 (6.9)	1631 (35.1)	54 (5.5)
	Other	75 (0.8)	21 (0.6)	31 (0.7)	23 (2.3)
**Age (years), n (%)**					
	<35	1882 (20.4)	739 (20.5)	958 (20.6)	185 (18.7)
	35‐44	2403 (26)	833 (23.1)	1224 (26.4)	346 (35)
	45‐54	2529 (27.3)	1108 (30.7)	1161 (25)	260 (26.3)
	>54	2426 (26.3)	930 (25.7)	1297 (28)	199 (20)
**Working sector, n (%)**					
	Public	7659 (82.3)	3115 (86.3)	3698 (79.7)	846 (85.5)
	Private or other	1581 (17.7)	495 (13.7)	942 (20.3)	144 (14.5)
**Participation in HIS^c^ or CIS^d^ development, n (%)**					
	Yes, I have been given time for it	480 (5.2)	216 (6)	202 (4.3)	62 (6.3)
	Yes, in addition to my work	1758 (19)	580 (16)	914 (19.7)	264 (26.7)
	No	7002 (75.8)	2814 (78)	3524 (76)	664 (67)
**Self-rated stress, n (%)**					
	No	3299 (35.7)	1037 (28.7)	1830 (39.4)	432 (43.6)
	Yes	5941 (64.3)	2573 (71.3)	2810 (60.6)	558 (56.4)
Stress related to information systems, mean (SD**)**^e^		—^f^	3.7 (1.13)	3.5 (1.07)	3.06 (0.97)
Satisfaction with software providers’ development work, mean (SD)^g^		—^f^	2.56 (1.07)	2.24 (1.02)	2.71 (0.86)

a RN: registered nurse.

b SWP: social welfare professional.

c HIS: health information systems.

d CIS: client information systems.

e Range 1‐6 (registered nurses) and range 1‐5 (physicians and social welfare professionals).

f Not applicable.

g Range 1‐5 (all professional groups).

### Main Effects on Professionals’ Self-Rated Stress

Table 2 shows the results of logistic regression analyses. Professional group and satisfaction with software providers’ development work were both associated with self-rated stress. Physicians and SWPs had significantly lower odds of self-rated stress than RNs. In addition, higher satisfaction with software providers’ development work was associated with lower odds of self-rated stress.

**Table 2.** The results of logistic regression analysis for self-rated stress.

Variables		Univariable model		Fully adjusted model^a^	
		OR^b^ (95% CI^c^)	*P* value	OR^b^ (95% CI)	*P* value
**Professional group**					
	Registered nurses	1	–^d^	1	–^d^
	Physicians	–0.48 (–0.57 to –0.38)	<.001	–0.47 (–0.57 to –0.36)	<.001
	Social welfare professionals	–0.65 (–0.80 to –0.51)	<.001	–0.68 (–0.83 to –0.53)	<.001
**Participation in HIS^e^ or CIS^f^ development**					
	Yes, I have been given time for it	1	–^d^	1	–^d^
	Yes, in addition to my own work	0.07 (–0.14 to –0.28)	.50	0.11 (–0.11 to –0.32)	.32
	No	.008 (–0.19 to –0.20)	.93	–0.06 (–0.26 to –0.14)	.56
	Satisfaction with software providers’ development work^b^	–0.19 (–0.23 to –0.14)	<.001	–0.23 (–0.28 to –0.19)	<.001

a Adjusted for gender, age, working sector, professional group, participation in health information systems or client information systems development and satisfaction with software providers’ development work; *R*2 (McFadden)=0.03.

b The odds ratio represents the odds ratio for one unit change in the continuous independent variable, indicating the odds for passing from low stress to high stress.

c Confidence interval describes the amount of uncertainty associated with a sampling method.

d Not applicable (reference group).

e HIS: health information systems.

f CIS: client information systems.

### Main Effects on Professionals’ Stress Related to Information Systems

The results of linear regression analyses are shown in Table 3. Professional group, participation in HIS or CIS development, and satisfaction with software providers’ development work were all associated with SRIS. In the fully adjusted model, physicians had a significantly lower likelihood of SRIS than RNs. Furthermore, the professionals who did not participate in the HIS or CIS development work had a significantly lower likelihood of SRIS. Moreover, professionals had significantly lower likelihood of SRIS if they were satisfied with the software providers’ development work.

**Table 3.** The results of linear regression analysis for stress related to information systems.

Variables		Univariable model		Fully adjusted model^a^	
		b^b^ (95% CI^c^)	*P *value	b^b^ (95% CI)	*P* value
**Professional group**					
	Nurses	0	^d^—	0	^d^—
	Physicians	–.002 (–0.05 to 0.04)	.92	–0.11 (–0.16 to –0.07)	<.001
	Social welfare professionals	–.005 (–0.08 to 0.06)	.89	.007 (–0.07 to 0.06)	.83
**Participation in HIS^e^ or CIS^f^ development**					
	Yes, I have been given time for it	0	^d^—	0	^d^—
	Yes, in addition to my own work	0.05 (–0.05 to 0.16)	.29	–0.02 (–0.11 to 0.07)	.65
	No	–0.06 (–0.16 to 0.03)	.18	–0.14 (–0.23 to –0.06)	<.001
	Satisfaction with software providers’ development work^b^	–0.35 (–0.37 to –0.33)	<.001	–0.36 (–0.38 to –0.34)	<.001

a Adjusted for gender, age, working sector, professional group, participation in health information systems or client information systems development and satisfaction with software providers’ development work; *R*2=0.15

b Regression coefficients represent the predicted change in the value of dependent variable for each one unit increase in the value of independent variable. Negative association means that when independent variables’ values increase, dependent variables’ values decrease.

c Confidence interval describes the amount of uncertainty associated with a sampling method.

d Not applicable.

e HIS: health information systems.

f CIS: client information systems,

### The Interactions

**Figure 1.**
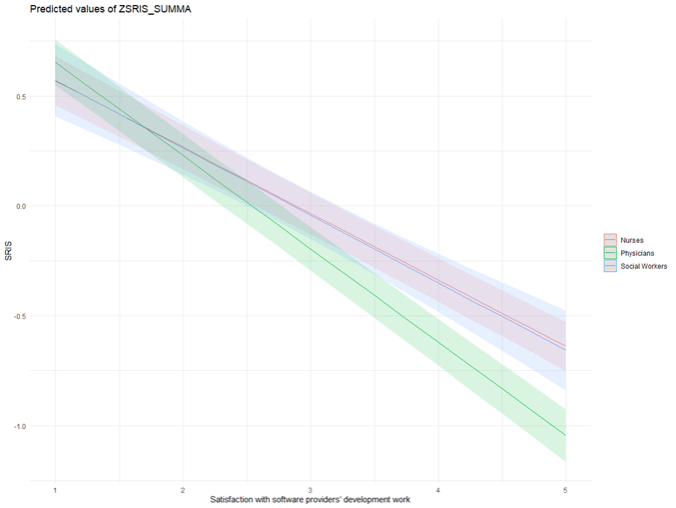
Interaction between professional groups and satisfaction with software provider’s development work for SRIS (the SRIS’s mean measure was standardized using *z* scores, where the value of the *z* score describes how many SDs you are away from the mean [mean always=0]).

There was a significant interaction effect between the professional group and satisfaction with software providers’ development work for SRIS. As can be seen in Figure 1, physicians had a significantly lower likelihood of SRIS *(*b=–0.12, 95% CI –0.16 to –0.08; *P*<.001) compared with RNs and SWPs if they were satisfied with software providers’ development work.

## Discussion

### Principal Findings

This study examined whether the factors that contribute to the successful implementation of information systems (participation in HIS or CIS development and satisfaction with software providers’ development work) are associated with well-being among RNs, physicians, and SWPs. More specifically, we examined whether there are differences between these professional groups, to gain valuable insights into the unique challenges and stressors faced by each professional group and highlight potential areas for improvement in IS development work. Our findings suggest that professional group, participation in HIS or CIS development, and satisfaction with software providers’ development work are significantly associated with professionals’ well-being and we found that there exist significant differences between these professional groups.

### Limitations

This study acknowledges some limitations. First, while the sample comprised 9240 health care professionals and SWPs, response rates were notably lower among RNs and SWPs, possibly affecting generalizability. However, structurally collected data correspond quite well to the overall composition of the professional groups, so it can be assumed that the results can be generalized with certain reservations. Furthermore, the response rate for SWPs may not be as reliable as desired because invitations to participate were sent via email and social media when the number of respondents was at risk of remaining low. Consequently, some respondents may have received the link from colleagues or via social media [37]. Second, caution is needed when extending findings to less digitally advanced countries, given Finland’s advanced digitalization [57]. Third, due to the cross-sectional nature of the study, causal relationships cannot be inferred. Fourth, while adjustments were made, unmeasured confounders like time pressure might have impacted results. Fifth, self-reported measures were used, not objective tests. Sixth, the ongoing personnel shortage and the COVID-19 pandemic during data collection might have influenced responses. Finally, the data collection took place at slightly different times, between March 2020 and February 2021.

### Comparison With Prior Work

The study revealed that RNs experienced higher levels of self-rated stress than physicians and SWPs. There are several factors that might contribute to the higher stress levels in nursing, such as the ongoing shortage of nurses in many countries, including Finland [19,24]. Furthermore, our findings reveal that physicians, whose SRIS has steadily increased in recent years [28] experienced significantly less SRIS than RNs. The higher level of SRIS among RNs may be explained by the common tendency for RNs to experience higher stress overall, as supported by the earlier findings [69,70]. Moreover, past literature has shown that the implementation of IS can be complex and time-consuming and requires RNs to invest significant effort in adapting new technologies to their workflows [15,16]. This can result in heightened stress levels among RNs [1,5]. Furthermore, a recent study found that ISs have led to a transfer of work tasks, specifically from physicians to RNs, resulting in an increased workload for RNs [71]. However, additional research is needed to validate these observations within the specific context of our study.

Participation in IS development work is an important factor for the success of IS implementations [6,72-74]. However, our findings indicate that professionals who did not participate in HIS or CIS development were experiencing less SRIS. Balancing the demands of daily work responsibilities while actively participating in IS development can be overwhelming and lead to stress for professionals. Participation in IS development work may lead to stress due to limited technical expertise or familiarity with a complex IS, leading to difficulties in understanding and effectively using the technology [6,14]. Furthermore, one possible explanation for higher stress levels among professionals is that those who possess digital competence and actively engage in IS development often have their roles emphasized and bear greater responsibility for ISs, resulting in a heavier workload compared with their peers [71].

Moreover, professionals often encounter stress as they navigate the challenges associated with change management, such as resistance from colleagues, uncertainty regarding the impact of changes, and potential disruptions to established routines [32,33]. Previous research indicates that organizations can facilitate professionals in allocating time for IS development work through various means. These include creating dedicated time slots for development, redistributing tasks, adjusting workload, and fostering a supportive work environment [37,38,75,76]. These strategies may help professionals engage more effectively in IS development activities while managing their regular work responsibilities.

Our study highlighted that dissatisfaction with software providers’ development work was associated with both self-rated stress and SRIS among professionals. Our findings can partially be explained by the previous studies conducted in Finland, where they found that only half of the professionals knew how and to whom to send their feedback, and only a few believed that vendors were genuinely interested in their viewpoints or that the suggested corrections and change requests were implemented within a reasonable time frame [35,37,38,45]. However, further research is needed to explore this aspect in greater depth. Prior research has highlighted the importance of effective collaboration between professionals and software providers, including responsiveness to feedback and the use of professionals’ input and expertise [6,37,38]. It has also been found that the lack of control and influence over decision-making processes, along with inadequate consideration of professionals’ viewpoints, can lead to frustration and stress [33]. For instance, SWPs often perceive that CISs are primarily developed from an administrative perspective to align with organizational objectives, thereby not fully incorporating professionals’ perspectives [43,44].

Our findings emphasize the importance of satisfaction with software providers’ development work, especially among physicians. Physicians had a significantly lower likelihood of SRIS compared with nurses and SWPs if they were satisfied with software providers’ development work. This may indicate that physicians value the efficient performance of software providers’ work within the context of the IS development process. This finding is a significant addition to prior research, which highlighted physicians’ high expectations from software provider, particularly regarding responsiveness to end-user feedback and implementation of corrections within a reasonable time frame [38]. Physicians’ active participation in HIS development work offers a sense of job control [10], which is crucial due to their primary responsibility for critical clinical decisions that directly affect patient care and outcomes. A smooth collaboration with the system provider ensures that the IS aligns with their decision-making needs, minimizing the risk of errors or delays in patient care [77].

### Conclusions

This study offers valuable insights into the factors impacting the successful implementation of ISs and their influence on the well-being of RNs, physicians, and SWPs. It emphasizes the importance of actively seeking and incorporating professional feedback, allocating dedicated time for HIS development, fostering collaborations with software providers, and addressing the unique needs of each professional group. In light of the nurse shortage, it is crucial to allocate dedicated time for HIS development, particularly for those involved in IS development, independently from patient care. Physicians can benefit from stronger collaboration with software providers, emphasizing feedback incorporation and job control through their involvement in HIS development. SWPSs’ unique needs should be prioritized in the development of CISs, going beyond administrative tasks and objectives. By addressing these factors, health care and social welfare organizations can enhance the well-being of professionals, improve the successful implementation of ISs, and ultimately enhance the quality of care and services provided to patients and clients. Future research should explore well-being among these professional groups in relation to other factors crucial for successful implementation, such as communication and training.

## Supplementary material

10.2196/52817 Multimedia Appendix 1 Measures used in the study.

## Abbreviations

CIS: client information system
HIS: health information system
IS: information system
OR: odds ratio
RN: registered nurse
SRIS: stress related to information systems
SWP: social welfare professional
WBSC: well-being service county
