# Supplementary material for: Factors Contributing to Successful Information System Implementation and Employee Well-Being in Health Care and Social Welfare Professionals: Comparative Cross-Sectional Study
Source: JMIR Med Inform. 2024 Nov 21;12:e52817. doi: 10.2196/52817 (PMC11604090; doi:10.2196/52817)
Supplement: Multimedia Appendix 1 [file medinform-v12-e52817-s001.docx]

**Multimedia Appendix**

**Measures used in the study**

**Registered nurses:**

**Dependent variables:**

***Stress related to information systems (SRIS):***

*How often have you been distracted, worried, or stressed (during the past half-year period)?*

- Constantly changing information systems
- Difficult, poorly performing IT equipment/software

Response options:

1. Never
2. Very rarely
3. Quite rarely
4. Quite often
5. Very often
6. Constantly

***Satisfaction with software providers’ development work***

*What kind of experiences have you had about providing feedback on the health care*

*information systems you use and about development? Please assess the following statements*

*based on your experiences.*

- The system vendor is interested in end users' feedback about the system
- The system vendor implements corrections and change requests according to the suggestions of the end users
- Corrections and change requests are implemented within a reasonable time frame

Response options:

1. Fully agree
2. Somewhat agree
3. Neither agree nor disagree
4. Somewhat disagree
5. Fully disagree
6. I cannot say

**Independent variables**

***Self-rated stress:***

*Stress means a situation where a person feels tense, restless, nervous or anxious or finds it hard*

*to sleep because they constantly worry about things. Do you feel such stress now?*

1. Not at all
2. Just a little
3. To some extent
4. Quite a lot
5. Very much

***Participation in HIS/CIS development:***

*Have you participated in information systems’ development work?*

1. Yes, some of my working time has been allocated for such development work
2. Yes, in addition to my work
3. No

**Control variables**

***Gender:***

1. Male
2. Female
3. Other
4. I don’t wish to say

***Year of birth (please select):***

[2003-1950]

***Main employment sector:***

1. Public (for example hospital district or municipality)
2. State
3. Private
4. University or other educational institution
5. Third sector

**Physicians**

**Dependent variables:**

***Stress related to information systems (SRIS):***

*How often has each of the issues mentioned below clearly disturbed, worried or burdened you at work during the past 6 months?*

- Changing EHR systems.
- Awkward, poorly functioning information technical equipment/software.

Response options:

1. Very often or constantly
2. Quite often
3. Every once in a while
4. Quite rarely
5. Very rarely or never

***Satisfaction with software providers’ development work***

*What kind of experiences have you had about providing feedback on the health care*

*information systems you use and about development? Please assess the following statements*

*based on your experiences.*

- The system vendor is interested in feedback about the system provided by the end users.
- The system vendor implements corrections and change requests according to the suggestions of the end users.
- Corrections and change requests are implemented within a reasonable time

Response options:

1. Fully agree
2. Somewhat agree
3. Neither agree nor disagree
4. Somewhat disagree
5. Fully disagree

**Independent variables**

***Self-rated stress:***

*Stress means a situation where a person feels tense, restless, nervous or anxious or finds it hard*

*to sleep because they constantly worry about things. Do you feel such stress now?*

1. Not at all
2. Just a little
3. To some extent
4. Quite a lot
5. Very much

***Participation in HIS/CIS development:***

*Have you participated in information systems development work?*

1. Yes, some of my working time has been allocated for such development work
2. Yes, in addition to my work
3. No

**Control variables**

***Gender:***

1. Male
2. Female
3. Other
4. I don’t wish to say

***Year of birth (please select):***

[1999-1956]

***Main employment sector:***

1. Municipality
2. State
3. Private (incl. The Social Insurance Institution of Finland (Kela))
4. University
5. I am not employed

**Social welfare professionals**

**Dependent variables**

***Stress related to information systems (SRIS):***

*How often have you been distracted, worried, or stressed (during the past half-year period)?*

- Constantly changing information systems
- Difficult, poorly performing IT equipment/software

Response options:

1. Very rarely or never
2. Quite rarely
3. Every now and then
4. Quite often
5. Very often or constantly

***Satisfaction with software providers’ development work***

*What kind of experiences have you had about providing feedback on the health care*

*information systems you use and about development? Please assess the following statements*

*based on your experiences.*

- The system vendor is interested in end users' feedback about the system
- The system vendor implements corrections and change requests according to the suggestions of the end users
- Corrections and change requests are implemented within a reasonable time frame

Response options:

1. Fully agree
2. Somewhat agree
3. Neither agree nor disagree
4. Somewhat disagree
5. Fully disagree

***Self-rated stress:***

*Stress means a situation where a person feels tense, restless, nervous or anxious or finds it hard*

*to sleep because they constantly worry about things. Do you feel such stress now?*

1. Not at all
2. Just a little
3. To some extent
4. Quite a lot
5. Very much

**Independent variables**

***Participation in HIS/CIS development:***

*Have you participated in information systems’ development work?*

1. Yes, some of my working time has been allocated for such development work
2. Yes, in addition to my work
3. No

**Control variables**

***Gender:***

1. Female
2. Male
3. Other
4. I don’t wish to say

***Age:***

1. Under 25
2. 25-34
3. 35-44
4. 45-54
5. 55-64
6. Over 64

***Main employment sector:***

1. Public
2. Private
3. Other (eg. associations) The Social Insurance Institution of Finland (Kela))
4. I canno’t say
